# Preoperative Surgical Fear and Association with Postoperative Pain and Quality of Recovery After Total Joint Arthroplasty

**DOI:** 10.3390/jcm15093451

**Published:** 2026-04-30

**Authors:** Kenan Gumus, Gülden Küçükakça Çelik, Özkan Öztürk

**Affiliations:** 1 Department of Surgical Nursing, Faculty of Health Sciences, Amasya University, Amasya 05200, Turkey; 2 Department of Surgical Nursing, Faculty of Health Sciences, Nevşehir Hacı Bektaş Veli University, Nevşehir 50300, Turkey; guldenkucukakca@hotmail.com; 3 Department of Orthopedic Trauma Surgery, Faculty of Medicine, Amasya University, Amasya 05200, Turkey; ozkan.ozturk@amasya.edu.tr

**Keywords:** knee arthroplasty, hip arthroplasty, fear, postoperative pain, recovery of function, perioperative care, orthopedic nursing

## Abstract

**Background:** Recovery following total joint arthroplasty varies substantially among patients, and psychological factors may partly account for this variability. Although anxiety and depression have been widely investigated, the specific contribution of preoperative surgical fear to postoperative pain and quality of recovery remains unclear. This study aimed to examine the association between preoperative surgical fear and postoperative pain intensity and quality of recovery. **Methods:** This prospective, hospital-based observational study enrolled 89 patients undergoing primary total knee or hip arthroplasty. Preoperative surgical fear was measured using the Surgical Fear Questionnaire (SFQ). Pain intensity was assessed with the Numeric Rating Scale (NRS) preoperatively and at three postoperative time points. Recovery quality at 24 h was evaluated using the Quality of Recovery-40 (QoR-40). Pearson correlation and multiple linear regression analyses were performed to evaluate associations and identify variables independently associated with recovery outcomes, controlling for potential confounders, including age, sex, ASA physical status, and type of surgery. **Results:** The mean SFQ score was 26.62 ± 15.19, and the mean QoR-40 score was 157.63 ± 16.66. Surgical fear was moderately and negatively correlated with overall recovery quality (r = −0.546, *p* < 0.001). In multiple linear regression analysis, surgical fear was most strongly associated with poorer overall recovery quality (β = −0.563, *p* < 0.001), within a model explaining 30.3% of the variance (adjusted R^2^ = 0.303). At the subscale level, surgical fear was significantly associated with emotional state, pain, physical comfort, and perceived support. Pain intensity at 12 h postoperatively was significantly associated with reduced physical independence (β = −0.218, *p* = 0.038). Pain intensity peaked at 12 h postoperatively (*p* < 0.001). **Conclusions:** Higher levels of preoperative surgical fear are associated with poorer quality of recovery following total joint arthroplasty. These findings highlight surgical fear as a potentially relevant perioperative factor and support the integration of routine psychological assessment into perioperative care pathways in relation to early postoperative recovery outcomes. From a clinical perspective, early identification of patients with high surgical fear may facilitate targeted perioperative counseling and supportive interventions by healthcare professionals, potentially improving recovery outcomes.

## 1. Introduction

Total arthroplasty surgery is among treatment methods that effectively improve the quality of life of individuals with end-stage joint disease [1,2]. Evidence suggests that, with the increasing elderly population, the number of patients undergoing surgery will increase by 40.14% for primary hip replacement surgery and 79.78% for primary knee replacement surgery by 2060 compared to 2024 [3]. Despite this growing surgical burden, most patients report positive postoperative outcomes, including improvements in pain, strength, and range of motion [1,2]. In a large sample study evaluating patient outcomes after hip and knee arthroplasty, it is stated that the proportion of satisfied patients was reported to be 90% and the rates of THA and TKA patients reporting very good joint status are 92.6% and 81.6%, respectively [4]. However, some patients still experience severe postoperative pain, which negatively affects the quality of recovery and increases the risk of delayed mobilization and prolonged hospitalization. Therefore, in recent years, the role of social and psychological factors contributing to suboptimal recovery outcomes has received considerable attention [5].

Among psychological factors, surgical fear is defined as an emotional response that begins when the patient’s surgery date is determined, and fear prior to surgery is considered a natural response [6]. However, numerous studies have shown that high levels of surgical fear across different types of surgery negatively affect the postoperative recovery process [7,8,9]. In orthopedic surgery, it is reported that the level of preoperative surgical fear ranges from 58% to 72% [10,11]. In addition, the current literature includes studies evaluating the association between psychological factors and changes in patient reported outcome measures after arthroplasty. These studies commonly assess the relationship between preoperative anxiety and depression and postoperative pain, function, and quality of recovery [1,2,12,13,14,15,16]. Some studies addressing fear in orthopedic surgery have examined fear and related factors [6], the relationship between fear and activities of daily living [17], the effect of fear of pain on postoperative pain intensity [18], and the relationship between mental well-being and surgical fear [19]. Studies evaluating quality of recovery have identified predictors affecting quality of recovery outcomes [20] and reported findings on the impact of pain catastrophizing and emotional distress on quality of recovery [21].

To the authors’ knowledge, limited evidence specifically addresses the combined association between preoperative surgical fear on both postoperative pain intensity and multidimensional quality of recovery in patients undergoing total knee and hip arthroplasty. Therefore, this study aims to address this gap by reviewing published arthroplasty studies and determine the association between patients’ preoperative surgical fear and postoperative pain intensity and quality of recovery outcomes.

Research Hypotheses

**H1:**  *Higher levels of preoperative surgical fear are associated with lower quality of recovery after total joint arthroplasty.*

**H2:**  *Higher levels of preoperative surgical fear are associated with increased postoperative pain intensity after total joint arthroplasty*.

**H3:**  *Increased postoperative pain intensity is associated with lower quality of recovery after total joint arthroplasty*.

## 2. Methods

### 2.1. Study Design and Setting

This prospective, hospital-based observational study was conducted to investigate the associational role of preoperative surgical fear on postoperative pain intensity and recovery quality. The research was carried out at the Orthopedics and Traumatology Clinic of Amasya University Training and Research Hospital between April 2024 and December 2025. The study reporting adhered strictly to the Strengthening the Reporting of Observational Studies in Epidemiology (STROBE) guidelines.

### 2.2. Participants

The study population consisted of patients scheduled for primary elective arthroplasty procedures. During the study period, a total of 131 patients who met the inclusion criteria were approached. Of these, 23 patients declined to participate, and 19 were excluded due to incomplete data (failure to fully complete the study forms). Consequently, the study was completed with 89 patients. A priori power analysis was conducted using G*Power 3.1 for multiple linear regression (fixed model, R^2^ deviation from zero). Assuming a medium effect size (f^2^ = 0.15), α = 0.05, and power = 0.80, the required minimum sample size was calculated as 88 participants. Thus, the final sample size exceeded the minimum required sample size.

Inclusion Criteria: Patients aged 18 years or older, scheduled for primary elective Total Knee Arthroplasty (TKA) or Total Hip Arthroplasty (THA) for the first time, classified under the American Society of Anesthesiologists (ASA) I-III physical status, and providing voluntary informed consent.

Exclusion Criteria: Individuals with prior history of joint replacement surgery [revision cases], cognitive impairments, emergency cases (e.g., trauma related hip fractures), diagnosed psychiatric disorders, or those who experienced severe intraoperative complications that precluded postoperative assessment. No patients undergoing additional concurrent surgical procedures were included.

### 2.3. Data Collection Instruments

#### 2.3.1. Patient Identification Form

This form was used to collect data on a total of 11 variables, including demographic characteristics (gender, marital status, and education level) and clinical parameters (previous surgery, duration of joint pain, ASA physical status, comorbidities, information received about surgery, time from admission to surgery, and types of surgery and anesthesia administered).

#### 2.3.2. The Surgical Fear Questionnaire (SFQ) 

The Surgical Fear Questionnaire (SFQ), developed by Theunissen et al. (2014) and validated for the Turkish population by Bağdigen and Karaman Özlü (2018), was used to assess preoperative surgical fear [22,23]. The instrument consists of 8 items rated on an 11-point numeric scale ranging from 0 (‘not at all afraid’) to 10 (‘extremely afraid’). It comprises two subscales: Short-Term Fear (SFQ-S; items 1–4) and Long-Term Fear (SFQ-L; items 5–8). Each subscale score ranges from 0 to 40, and the total score ranges from 0 to 80, where higher scores indicate greater surgical fear. In the present study, Cronbach’s alpha coefficient for the total scale was 0.89.

#### 2.3.3. The Numeric Rating Scale (NRS)

The Numeric Rating Scale (NRS) is a unidimensional measure of pain intensity widely recognized for its clinical validity and applicability in acute postoperative settings [24]. In this study, it was used to quantify patients’ subjective pain experience. The scale consists of an 11-point numeric scale ranging from 0 (“no pain”) to 10 (“worst imaginable pain”). Pain intensity was recorded preoperatively (NRS-T0), immediately before the first postoperative analgesic administration (NRS-T1), which corresponds to the first postoperative clinical assessment when analgesia was required, and at the 12th (NRS-T2) and 24th (NRS-T3) postoperative hours.

#### 2.3.4. Quality of Recovery-40 (QoR-40)

Quality of Recovery-40 developed by Myles et al. (2000) [25] and validated in Turkish by Karaman et al. (2014) [26], was used to assess postoperative recovery quality. The questionnaire consists of 40 items across five dimensions: emotional state [9 items], physical comfort (12 items), patient support (7 items), physical independence (5 items), and pain (7 items). Each item is rated on a 5-point Likert scale. Total scores range from 40 to 200, with higher scores indicating better recovery quality. In the present study, the Cronbach’s alpha coefficient for the total scale was 0.86 [25,26].

### 2.4. Data Collection Procedure

Eligible patients were identified and approached on the morning of their scheduled surgery. After obtaining written informed consent, the patient identification form, SFQ, and baseline pain intensity (NRS-T0) were administered. No sedative or anxiolytic premedication was administered prior to SFQ completion; only standard prophylactic antibiotic treatment was given according to routine clinical protocol. Postoperative pain intensity was assessed at NRS-T1 and subsequently at NRS-T2 and NRS-T3. All patients received a standardized institutional multimodal analgesia protocol. No patient controlled analgesia or regional anesthesia techniques were used as part of the postoperative analgesic management.

NRS-T1 was recorded during the first postoperative clinical assessment on the ward, immediately before the administration of the first postoperative analgesic. This assessment was performed after transfer from the recovery unit, at a mean interval of approximately 60–90 min following completion of surgery, depending on individual recovery and transfer timing.

Following NRS-T1 assessment, all patients received 1 g intravenous acetaminophen. Intravenous tenoxicam (20 mg) was administered 12 h postoperatively as part of the routine multimodal analgesic regimen. Rescue analgesia with 100 mg intravenous tramadol was administered when pain intensity exceeded NRS > 4, and only after completion of scheduled pain assessments at each time point to avoid interference with outcome measurements. All patients were managed under the same standardized institutional protocol.

The QoR-40 questionnaire was administered at the 24th postoperative hour to evaluate recovery quality. This 24 h postoperative time point was chosen in accordance with the validated use of the QoR-40 instrument in the literature for early postoperative recovery assessment [27]. The preoperative and postoperative questionnaires were administered by the same researchers; therefore, outcome assessors were not blinded to participants’ preoperative fear scores. Each assessment session was completed in approximately 10–15 min.

### 2.5. Statistical Analysis

Data were analyzed using IBM SPSS Statistics Version 22.0 (IBM Corp., Armonk, NY, USA). After exclusion of non-participating and incomplete cases, all 89 patients included in the final analysis had complete datasets for all study variables. Therefore, no missing data were present in the analytical dataset, and all statistical analyses were conducted on complete cases without the need for imputation. Descriptive statistics were presented as frequencies, percentages, means, and standard deviations. Normality was assessed using skewness and kurtosis coefficients (±2). Independent samples *t*-tests and one-way ANOVA were used for group comparisons, followed by LSD or Dunnett’s C post hoc tests according to variance homogeneity. Pearson correlation analysis examined associations between variables. Multiple linear regression analyses were conducted to determine the associations of surgical fear and pain intensity with quality of recovery and its subdimensions. Potential confounders including age, sex, ASA status, and type of surgery were controlled for in the regression models. Regression assumptions were evaluated prior to analysis; multicollinearity was assessed using tolerance and variance inflation factor (VIF) values (VIF < 5, tolerance > 0.20). The assumptions of multiple linear regression, including normality of residuals, linearity, and homoscedasticity, were evaluated and no significant violations were observed. Statistical significance was *p* < 0.05.

### 2.6. Ethical Considerations

Ethical approval was obtained from the Amasya University Non Interventional Clinical Research Ethics Committee (Approval No: 2024/31; Date: 25 March 2024). Institutional permission was granted by the hospital administration. The study was conducted in accordance with the principles of the Declaration of Helsinki. Written informed consent was obtained from all participants prior to data collection.

## 3. Results

Comparisons of scale scores according to demographic and clinical characteristics are presented in Table 1. The majority of the patients were female (83.1%), married (84.3%), and had completed primary education (48.3%). Regarding clinical characteristics, 73.0% of the participants had previous surgical experience, 38.2% had been experiencing joint pain for 2–6 years, and 60.7% were classified in the ASA physical status II risk group. In addition, 92.1% of the patients reported being informed about the surgical process, 87.6% had an interval of 12–24 h between hospital admission and surgery, and 77.5% had comorbidities. With respect to operative characteristics, 74.2% of the patients underwent TKA, and spinal anesthesia was administered to 89.9% of the participants.

Statistical analyses demonstrated that gender was significantly associated with the quality of recovery. The mean QoR-40 score was higher in male patients compared to female patients (*p* = 0.003). A statistically significant difference was identified between previous surgical history and postoperative pain levels, as measured by NRS-T1 (*p* = 0.025) and NRS-T3 (*p* < 0.001). Patients without a history of surgery had lower NRS-T1 scores, whereas their NRS-T3 scores were higher than those of patients with a previous surgical history. In the evaluation of ASA physical status classification, a significant difference was observed only in SFQ scores (*p* = 0.046), with patients classified as ASA I reporting higher fear scores compared to the other groups. In comparisons based on the type of surgery, SFQ scores were higher among patients who underwent Total Hip Arthroplasty (THA) (*p* = 0.035), while patients in the TKA group had higher NRS-T3 scores (*p* = 0.025).

The distribution of the scores obtained by the participants from the surgical fear, pain intensity, and quality of recovery scales and their subscales is presented in Table 2. The mean SFQ score was 26.62 ± 15.19 (SFQ-S: 18.06 ± 10.59; SFQ-L: 8.70 ± 5.75), and the mean QoR-40 score was 157.63 ± 16.66. When the QoR-40 subscales were examined, patients achieved the highest mean score in the physical comfort subscale (45.92 ± 6.43) and the lowest mean score in the physical independence subscale (15.23 ± 3.88). Regarding pain intensity, the mean NRS score was 5.40 ± 2.98 at T0, 4.53 ± 2.85 at T1, 6.42 ± 2.15 at T2, and 4.71 ± 1.98 at T3.

The results of the correlation analyses between surgical fear, pain intensity, and quality of recovery scales and their subscales are presented in Table 3. A negative, moderate, and statistically significant correlation was identified between SFQ scores and QoR-40 scores (r = −0.546; *p* < 0.001). At the subscale level, surgical fear demonstrated significant negative correlations with emotional state, pain, patient support, and physical comfort (*p* < 0.001, *p* < 0.001, *p* < 0.001, and *p* = 0.002, respectively). The SFQ-S and SFQ-L subscales were also significantly and negatively correlated with the QoR-40 total score (*p* < 0.001 for both).

Regarding the relationships between pain intensity and quality of recovery, NRS-T2 (*p* = 0.006) and NRS-T3 (*p* < 0.001) scores were found to be significantly and negatively correlated with the physical independence subscale.

According to the data presented in Table 4, which examine the temporal changes in participants’ pain intensity scores, the differences among measurement time points were statistically significant (F = 9.725; *p* <0.001). The analysis indicated that this change in pain scores was associated with a moderate effect size (partial eta squared = 0.100).

**Figure 1:**
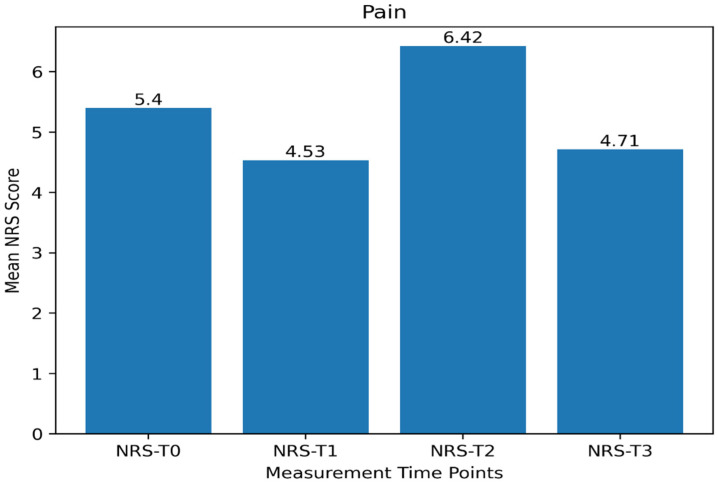
Distribution of Pain Intensity According to Measurement Time Point.

Examination of the temporal trend in mean pain scores (Figure 1) revealed that the NRS-T2 score was significantly higher than both the NRS-T1 and NRS-T3 scores. These findings indicate that patients experienced the highest level of pain at the 12th postoperative hour.

Multiple regression models examining the associations of surgical fear and pain intensity with quality of recovery are presented in Table 5. The model for the total QoR-40 score explained 30.3% of the total variance (adjusted R^2^ = 0.303; F = 8.663; *p* < 0.001), indicating a moderate level of explained variability with a substantial proportion of variance remaining unexplained. In this context, surgical fear was the only variable significantly associated with QoR-40 total scores, with a negative association indicating poorer recovery outcomes (Beta = −0.563; *p* < 0.001).

At the subscale level, surgical fear emerged as the primary variable associated with emotional state and pain subdimensions, with the model explaining 23.4% and 19.3% of the variance, respectively (Beta = −0.488 and Beta = −0.475; *p* < 0.001). Similarly, surgical fear was predominantly negatively associated with on physical comfort (Beta = −0.364) and patient support (Beta = −0.422; *p* < 0.01). In the physical independence model, the effect of surgical fear remained at the threshold of statistical significance (*p* = 0.054), whereas NRS-T2 pain intensity was identified as a significant association, explaining 17.4% of the variance in this dimension (adjusted R^2^ = 0.174; Beta =−0.218; *p* = 0.038).

## 4. Discussion

This study examined the relationship between preoperative surgical fear, postoperative pain intensity, and quality of recovery. The findings suggest that higher levels of preoperative surgical fear may be associated with lower quality of postoperative recovery, and that increased postoperative pain intensity may be related to greater physical dependence during recovery.

### 4.1. Surgical Fear, Recovery, and Pain by Participant Characteristics

Preoperative emotional well-being affects both physiological and psychological recovery during the postoperative period. In particular, preoperative fear is a common emotional response among many patients awaiting surgery [28]. It is considered an anxiety-inducing factor in 60–80% of patients before surgery [29], and various factors may influence surgical fear [28]. Although the findings of the present study do not indicate a high overall level of surgical fear, surgical fear was found to be higher in patients with an ASA score of I and in those who had undergone TKA. Similarly, previous studies have shown poorer psychological well-being among orthopedic patients with ASA levels I/II [30] and among those undergoing TKA [6]. These findings suggest that preoperative fear, which is associated with the quality of recovery in orthopedic surgery, should be evaluated in conjunction with the type of surgery and ASA classification.

This study examines a patient population in orthopedic surgery with a good level of quality of recovery. The results indicate that men report better quality of recovery than women. In contrast, Wessels et al. (2021) reported that gender was not a significant factor affecting quality of recovery in orthopedic surgery [20]. Similarly, Allen and Zumwalt (2024) stated that men tended to achieve better surgical outcomes after procedures such as total joint arthroplasty [31]. Özdemir et al. (2022) [32] concluded that female patients may experience more sleep and activity disturbances, negative emotional experiences, and higher pain intensity compared to male patients, which may negatively affect quality of recovery. Therefore, they emphasized that pain intensity, in addition to sociodemographic characteristics, may be an important parameter influencing patients’ quality of recovery [32].

In the present study, postoperative pain intensity peaked at 12 h. However, pain intensity was higher before the first analgesic administration in patients with a history of previous surgery and at 24 h in patients without prior surgical experience. In contrast to the present findings, a study involving orthopedic patients reported more severe pain at 24 h than at 12 h postoperatively [33]. Another study of surgical patients showed that pain intensity was high within the first 24 h in patients undergoing orthopedic surgery and was even higher in those with previous surgical experience [34]. Conversely, a study of patients undergoing knee replacement surgery demonstrated lower postoperative pain levels at 6 h in patients with prior surgical experience [18]. These differences in findings may stem from patients’ previous pain experiences and pain perceptions. Indeed, the literature suggests that patients who have previously undergone painful surgical procedures may develop anxiety and fear regarding subsequent surgeries, and these emotional responses may exacerbate their perception of pain [35]. This finding may be explained by several clinical factors. One possible explanation is the waning effect of regional anesthesia, as the duration of single shot nerve blocks is generally limited and may decrease within the first 8–24 h depending on the anesthetic agent used and individual patient characteristics. As the effect of regional blockade diminishes, a rebound increase in pain intensity may occur during the early postoperative period. In line with this, it has been reported that the limited duration of peripheral nerve blocks may contribute to increased pain once analgesic effects wear off [36]. In addition, the timing and organization of postoperative analgesic protocols, including multimodal and opioid administration schedules, may contribute to fluctuations in pain intensity. Delays in initiating or optimizing postoperative analgesia during the transition from intraoperative to postoperative care can create temporary gaps in pain control, which may be reflected as increased pain scores in the early recovery phase [37].

### 4.2. Association of Surgical Fear and Pain with Recovery

The fear avoidance model of pain, detailed by Rogers and Farris [38], offers a psychosocial framework that integrates well with neurobiological insights. In this model, it is argued that hypervigilance and catastrophizing of pain stimulate limbic circuits and stress responses, thereby strengthening central sensitivity. Patients who develop increased fear responses to pain may reduce their activity levels, leading to deconditioning, social withdrawal, and exacerbation of psychological distress. Therefore, neurocognitive mechanisms can act as a bridge between psychological risk factors and biological changes, negatively impacting the patient’s recovery process. In this context, these relationships provide a strong clinical rationale for routine psychosocial assessments in orthopedic surgery.

In orthopedic surgery patients, postoperative pain and recovery may be associated with emotional states such as anxiety and fear [39]. Similarly, the findings of the present study indicate that preoperative fear levels are associated with indicators of recovery quality, including physical comfort, emotional state, perceived support in care, and increased pain intensity. Furthermore, preoperative surgical fear was negatively associated with these parameters, reflecting poorer recovery outcomes in patients. The magnitude of the association suggests that surgical fear may be related to a proportion of the variability in early recovery. A plausible explanation is that elevated surgical fear may heighten pain perception through increased attentional focus on bodily sensations and stress related physiological activation. In this context, stress induced neuroendocrine responses may contribute to enhanced inflammatory activity and greater pain sensitivity in the acute postoperative period [40]. In addition, fear related cognitive patterns such as catastrophizing and avoidance may reduce engagement in early mobilization, thereby influencing multiple domains of recovery quality.

Khalil et al. (2025) reported that emotional distress was a negative determinant of recovery quality in orthopedic surgery patients [21], while Çelik et al. (2024) stated that surgical fear was a significant factor influencing the spiritual well-being of patients with gonorrhea [19]. Similarly, managing patients’ preoperative anxiety has been emphasized as necessary to minimize negative postoperative outcomes, such as inadequate recovery and increased analgesic requirements [9]. Wei et al. (2025) underlined that fear plays a critical role in the rehabilitation process of orthopedic surgery patients [41]. High preoperative anxiety levels were identified as predictors of recovery quality both 3 days postoperatively [12] and even 6 months postoperatively [42]. Therefore, it is beneficial for a multidisciplinary team of healthcare professionals, such as orthopedists, physiotherapists, and nurses, to consider the impact of these psychological factors, include them in preoperative patient assessment parameters, and evaluate them using reliable measurement tools, in order to improve the quality of the patient’s recovery outcomes.

It has been reported that patients with higher preoperative anxiety levels experience increased postoperative pain, decreased functionality, and more complications, indicating poorer recovery quality [1]. Additionally, emotional states such as anxiety and depression have been associated with acute postoperative pain in orthopedic surgery patients [5]. Patients with high levels of surgical fear have been shown to experience greater pain and more physical difficulty during movement [9]. One study emphasized that elevated anxiety levels may contribute to increased early postoperative pain intensity [2]. Psychological factors have also been associated with increased pain, which in turn is linked to decreased functional outcomes [1]. Consistent with the literature, another finding of the present study indicates a relationship between early postoperative pain intensity and physical dependence, with pain intensity at 12 h postoperatively being significantly associated with physical dependence. Qualitative studies involving orthopedic surgery patients have shown that pain during physical movement is perceived as a significant barrier, limiting participation in activities of daily living. Patients have stated that even if pain does not completely resolve, a reduction in its intensity improves their quality of life [17,43]. A quality assessment study encompassing 26 studies examining the effects of psychological factors on patient outcomes after TKA and THA found that the studies provided reliable results and offered reliable assessments of various psychological variables’ impact on patient recovery outcomes. However, a variable such as surgical fear was not evaluated in this comprehensive study [44]. Therefore, although the influence of psychological factors is known, it is clear that there is a need for comprehensive studies evaluating the impact of surgical fear on outcomes after TKA and THA. Therefore, these results support the notion that assessing and managing preoperative surgical fear may be important in relation to patients’ physical and emotional recovery outcomes.

While these findings provide clinically relevant insights, several limitations should be considered when interpreting the results. It was conducted at a single center and reflects data from one institutional database, which may limit generalizability to other populations or clinical settings. In addition, the study sample was predominantly female (83.1%), which may limit the applicability of the findings to male patients and should be considered when interpreting the results. Only short-term postoperative outcomes were evaluated (24 h), and medium- and long-term recovery trajectories were not assessed, which restricts conclusions regarding sustained recovery patterns.

Potential influencing factors such as disease severity were not included in the analyses. Furthermore, several potentially important confounding psychological variables, including subclinical baseline anxiety and depressive symptoms, pain catastrophizing, preoperative psychological distress, and baseline pain sensitivity, as well as other unmeasured factors, such as potential neurological or social conditions, were not measured in this study and therefore could not be included in the regression models. Although the SFQ was used as a total score in this study, it consists of two subdimensions (short-term and long-term fear), which may have differential predictive or associative value for postoperative outcomes. The present analysis did not examine these subscales separately; therefore, potential differences in their relationships with pain and recovery outcomes could not be evaluated.

In addition, 92.1% of patients reported being informed about the surgical procedure, which may introduce a potential selection related effect and limit variability in preoperative psychological responses. Only patients who underwent successful total knee and hip replacement surgery without major complications were included, which may also have introduced a selection bias. Finally, outcome assessment was performed by the same researchers who administered both preoperative and postoperative questionnaires, which may have introduced measurement bias.

The findings indicate that preoperative surgical fear is closely associated with early postoperative recovery quality in patients undergoing total joint arthroplasty, demonstrating consistent relationships across emotional, physical, and pain related recovery domains. These results suggest that recovery in the early postoperative period is associated not only with surgical and analgesic factors but also with preoperative psychological status.

Routine assessment of surgical fear using brief and validated instruments, such as the SFQ, which can be completed in approximately 2–3 min during preoperative nursing assessment, may assist perioperative clinicians in identifying patients at risk of poorer early recovery. Incorporating psychological evaluation into standard preoperative assessment may contribute to more individualized perioperative care planning. In addition, the observation that postoperative pain intensity peaked at 12 h and was associated with reduced physical independence highlights a clinically relevant period for intensified pain monitoring and supportive interventions. Given the high volume of total joint arthroplasty procedures worldwide, integrating psychological screening into perioperative pathways may contribute to improved early recovery outcomes.

## 5. Conclusions

Patients who underwent total knee and hip replacement surgery and exhibited relatively low levels of surgical fear and good recovery quality nevertheless reported high pain intensity, particularly at 12 h postoperatively. Surgical fear levels were higher among patients classified as ASA I and those who underwent total knee arthroplasty [TKA], and recovery quality was better among men. Additionally, patients with prior surgical experience demonstrated higher postoperative pain intensity scores.

Preoperative surgical fear was identified as significantly associated with both physical and emotional recovery quality. Moreover, increased postoperative pain intensity was significantly associated with physical dependence in patients. These findings highlight the importance of integrating psychological assessment into routine perioperative care protocols in orthopedic surgery settings.

## Figures and Tables

**Table 1:** Comparison of Surgical Fear, Pain, and Recovery Scores by Participant Characteristics.

		n	%	SFQ		NRS-T0		NRS-T1		NRS-T2		NRS-T3		QoR-40	
				X	SD	X	SD	X	SD	X	SD	X	SD	X	SD
Gender	Male	15	16.9	20.67	14.53	4.27	3.13	3.47	2.33	6.60	1.35	5.33	1.95	169.20	10.83
	Female	74	83.1	27.82	15.13	5.64	2.92	4.74	2.91	6.38	2.29	4.58	1.98	155.28	16.70
	*Test value*, *p*			t = −1.681 *p* = 0.096		t = −1.637 *p* = 0.105		t = −1.594 *p* = 0.115		t = 0.505 *p* = 0.617		t = 1.345 *p* = 0.182		t = 3.090 *p* = **0.003**	
Marital status	Single	14	15.7	32.79	14.11	4.93	3.43	5.64	2.47	6.43	1.55	4.57	1.91	150.00	14.30
	Married	75	84.3	25.47	15.20	5.49	2.91	4.32	2.89	6.41	2.25	4.73	2.01	159.05	16.76
	*Test value*, *p*			t = 1.671 *p* = 0.098		t = −0.649 *p* = 0.518		t = 1.607 *p* = 0.112		t = 0.031 *p* = 0.975		t = −0.279 *p* = 0.781		t = −1.894 *p* = 0.062	
Education level	Illiterate	10	11.2	16.70	12.36	4.70	3.53	3.20	2.94	6.40	3.20	3.70	1.77	160.40	16.79
	Literate	13	14.6	25.92	15.31	6.00	2.65	4.00	2.77	6.46	1.90	4.31	2.18	157.00	17.45
	Primary Education	43	48.3	27.00	16.75	5.63	3.21	4.49	2.85	6.21	2.09	4.86	1.87	159.42	17.48
	High school	17	19.1	30.47	12.96	5.12	2.69	5.71	2.82	7.06	1.78	5.24	2.25	153.47	13.86
	University or higher	6	6.7	31.00	7.62	4.50	1.87	4.83	2.56	6.00	2.37	4.67	1.75	153.33	18.45
	*Test value*, *p*			F = 1.511 *p* = 0.206		F = 0.496 *p* = 0.739		F = 1.424 *p* = 0.233		F = 0.524 *p* = 0.718		F = 1.150 *p* = 0.339		F = 0.551 *p* = 0.699	
Previous surgery	No	24	27.0	26.54	10.69	5.13	2.07	3.42	2.72	6.88	2.33	5.96	1.57	154.00	12.35
	Yes	65	73.0	26.65	16.62	5.51	3.26	4.94	2.81	6.25	2.08	4.25	1.93	158.97	17.89
	*Test value*, *p*			t = −0.035 *p* = 0.972		t = −0.654 *p* = 0.515		t = −2.286 *p* = **0.025**		t = 1.227 *p*= 0.223		t = 3.893 ***p* < 0.001**		t = −1.480 *p* = 0.144	
Duration of joint pain (Years)	≤1	6	6.7	19.50	19.90	7.00	3.22	4.33	2.50	7.17	1.72	6.00	1.79	162.50	17.48
	2–6	34	38.2	28.53	16.36	4.62	3.06	4.59	2.97	6.09	2.42	5.03	2.21	159.68	18.33
	7–12	33	37.1	25.79	12.23	5.94	2.69	4.33	2.85	6.76	1.87	4.42	1.70	155.27	14.98
	>12	16	18.0	26.94	16.86	5.38	3.10	4.88	2.96	6.13	2.25	4.13	1.93	156.31	16.57
	*Test value*, *p*			F = 0.646 *p* = 0.588		F = 1.763 *p* = 0.160		F = 0.140 *p* = 0.936		F = 0.877 *p* = 0.456		F = 1.886 *p* = 0.136		F = 0.587 *p* = 0.625	
ASA physical status	1	9	10.1	38.33	22.10	6.44	2.60	4.78	3.42	5.89	2.47	4.89	1.83	152.00	16.51
	2	54	60.7	25.70	13.87	5.46	3.14	4.67	2.85	6.72	1.84	4.91	1.99	158.89	16.73
	3	26	29.2	24.46	13.86	4.92	2.76	4.15	2.72	5.96	2.58	4.23	2.01	156.96	16.77
	*Test value*, *p*			F = 3.186 *p* = **0.046**		F = 0.896 *p* = 0.412		F = 0.317 *p* = 0.729		F = 1.409 *p* = 0.250		F = 1.064 *p* = 0.350		F = 0.684 *p* = 0.507	
Information received about surgery	No	7	7.9	21.00	15.45	5.57	3.60	5.43	2.44	6.57	2.15	3.86	1.35	165.86	18.83
	Yes	82	92.1	27.10	15.17	5.39	2.95	4.45	2.89	6.40	2.17	4.78	2.02	156.93	16.40
	*Test value*, *p*			t = −1.019 *p* = 0.311		t = 0.154 *p* = 0.878		t = 0.869 *p* = 0.387		t = 0.198 *p* = 0.843		t = −1.185 *p* = 0.239		t = 1.368 *p* = 0.175	
Comorbidity	No	20	22.5	31.70	18.86	5.35	3.03	5.00	2.58	6.40	2.21	4.60	2.06	154.50	16.90
	Yes	69	77.5	25.14	13.77	5.42	2.99	4.39	2.93	6.42	2.15	4.74	1.98	158.54	16.60
	*Test value*, *p*			t = 1.718 *p* = 0.089		t = −0.092 *p* = 0.927		t = 0.839 *p* = 0.404		t = −0.037 *p* = 0.971		t = −0.275 *p* = 0.784		t = −0.954 *p* = 0.343	
Time from admission to surgery (hours)	12–24	78	87.6	26.10	15.23	5.31	3.11	4.62	2.80	6.41	2.09	4.76	2.01	158.42	16.84
	25–36	7	7.9	35.00	13.98	5.43	1.81	3.14	3.08	6.71	3.04	4.14	1.68	146.57	5.80
	37–48	4	4.5	22.00	14.99	7.25	0.50	5.25	3.69	6.00	2.16	4.75	2.22	161.50	21.63
	*Test value*, *p*			F = 1.304 *p* = 0.277		F = 0.805 *p* = 0.450		F = 0.990 *p* = 0.376		F = 0.139 *p* = 0.870		F = 0.303 *p* = 0.739		F = 1.769 *p* = 0.177	
Type of surgery	Total Knee Arthroplasty	66	74.2	24.62	14.06	5.55	3.06	4.26	2.97	6.38	2.19	4.98	1.94	158.35	16.05
	Total Hip Arthroplasty	23	25.8	32.35	17.13	5.00	2.76	5.30	2.36	6.52	2.09	3.91	1.93	155.57	18.52
	*Test value*, *p*			t = −2.143 *p* = **0.035**		t = 0.754 *p* = 0.453		t = −1.705 *p* = 0.095		t = −0.273 *p* = 0.786		t = 2.284 *p* = **0.025**		t = 0.688 *p* = 0.493	
Type of anesthesia	General anesthesia	9	10.1	29.67	23.23	6.22	2.33	5.89	3.72	7.11	2.15	5.44	2.01	158.22	17.22
	Spinal anesthesia	80	89.9	26.28	14.19	5.31	3.04	4.38	2.73	6.34	2.15	4.63	1.98	157.56	16.70
	*Test value*, *p*			t = 0.633 *p* = 0.529		t = 0.867 *p* = 0.388		t = 1.521 *p* = 0.132		t = 1.023 *p* = 0.309		t = 1.177 *p* = 0.242		t = 0.112 *p* = 0.911	

t: Independent samples *t*-test, F: Analysis of variance (ANOVA), n: Number, X: Mean, SD: Standard deviation, SFQ: Surgical Fear Questionnaire, QoR-40: Quality of recovery-40 questionnaire, NRS-T0: Preoperative pain intensity, NRS-T1: Pain intensity measured immediately before the first postoperative analgesic administration, at the first postoperative clinical assessment when the patient first required analgesia, NRS-T2: Pain intensity at 12 h postoperatively, NRS-T3: Pain intensity at 24 h postoperatively.

**Table 2:** Descriptive Statistics of Surgical Fear, Pain Intensity, and Quality of Recovery-40 Scores.

Scales and Dimensions	n	Min.	Max.	X	SD
SFQ-S	89	0	50	18.06	10.59
SFQ-L	89	0	30	8.70	5.75
SFQ	89	0	80	26.62	15.19
Physical comfort	89	24	57	45.92	6.43
Emotional state	89	20	45	35.74	5.61
Physical independence	89	7	25	15.23	3.88
Patient support	89	21	35	31.85	3.46
Pain	89	16	35	28.11	3.65
QoR-40 total score	89	112	194	157.63	16.66
NRS-T0	89	0	10	5.40	2.98
NRS-T1	89	0	10	4.53	2.85
NRS-T2	89	0	10	6.42	2.15
NRS-T3	89	0	8	4.71	1.98

SFQ-S: Short-term Consequences; SFQ-L: Long-term Consequences; QoR-40: Quality of recovery-40 questionnaire; NRS-T0: Preoperative pain intensity, NRS-T1: Pain intensity measured immediately before the first postoperative analgesic administration, at the first postoperative clinical assessment when the patient first required analgesia, NRS-T2: Pain intensity at 12 h postoperatively, NRS-T3: Pain intensity at 24 h postoperatively, n: Number, Min-Max: Minimum–Maximum, X: Mean, SD: Standard deviation.

**Table 3:** Correlation Matrix of Surgical Fear, Pain Intensity, and Quality of Recovery.

		NRS-T0	NRS-T1	NRS-T2	NRS-T3	Physical Comfort	Emotional State	Physical Independence	Patient Support	Pain	QoR40
*SFQ-S*	r	0.114	0.138	−0.009	−0.072	−0.332	−0.521	−0.082	−0.403	−0.445	−0.529
	p	0.292	0.198	0.937	0.506	**0.002**	**<0.001**	0.448	**<0.001**	**<0.001**	**<0.001**
	n	89	89	89	89	89	89	89	89	89	89
*SFQ-L*	r	−0.105	0.263	−0.102	0.175	−0.261	−0.394	−0.251	−0.312	−0.397	−0.463
	p	0.331	**0.013**	0.346	0.102	**0.014**	**<0.001**	**0.018**	**0.003**	**<0.001**	**<0.001**
	n	89	89	89	89	89	89	89	89	89	89
*SFQ*	r	0.034	0.200	−0.042	0.029	−0.329	−0.512	−0.152	−0.398	−0.460	−0.546
	p	0.751	0.061	0.694	0.787	**0.002**	**<0.001**	0.158	**<0.001**	**<0.001**	**<0.001**
	n	89	89	89	89	89	89	89	89	89	89
NRS-T0	r	1	−0.107	−0.009	−0.228	0.038	−0.077	0.208	0.030	−0.113	−0.008
	p		0.318	0.935	0.032	0.725	0.473	0.052	0.781	0.293	0.939
	n	89	89	89	89	89	89	89	89	89	89
NRS-T1	r	−0.107	1	−0.073	−0.077	0.055	−0.166	0.128	0.000	0.002	−0.033
	p	0.318		0.496	0.474	0.612	0.122	0.234	0.999	0.987	0.758
	n	89	89	89	89	89	89	89	89	89	89
NRS-T2	r	−0.009	−0.073	1	0.319	−0.037	0.035	−0.289	−0.028	−0.093	−0.165
	p	0.935	0.496		0.002	0.731	0.748	**0.006**	0.792	0.387	0.122
	n	89	89	89	89	89	89	89	89	89	89
NRS-T3	r	−0.228	−0.077	0.319	1	0.031	−0.064	−0.337	0.011	−0.044	−0.147
	p	0.032	0.474	0.002		0.775	0.551	**0.001**	0.918	0.686	0.168
	n	89	89	89	89	89	89	89	89	89	89

r: Correlation coefficient, p: Significance, n: Number; SFQ: Surgical Fear Questionnaire; SFQ-S: Short-term Consequences; SFQ-L: Long-term Consequences; NRS-T0: Preoperative pain intensity, NRS-T1: Pain intensity measured immediately before the first postoperative analgesic administration, at the first postoperative clinical assessment when the patient first required analgesia, NRS-T2: Pain intensity at 12 h postoperatively, NRS-T3: Pain intensity at 24 h postoperatively.

**Table 4:** Temporal Changes in Pain Intensity Scores: Repeated Measures Analysis.

	NRS-T0		NRS-T1		NRS-T2		NRS-T3	
	X	SD	X	SD	X	SD	X	SD
Pain	5.40	2.98	4.53	2.86	6.42	2.15	4.71	1.99
Test value, *p*	F = 9.725; ***p* < 0.001**							
Partial η^2^	0.100							
Bonferroni	NRS-T2 > NRS-T1; NRS-T3							

NRS-T0: Preoperative pain intensity, NRS-T1: Pain intensity measured immediately before the first postoperative analgesic administration, at the first postoperative clinical assessment when the patient first required analgesia, NRS-T2: Pain intensity at 12 h postoperatively, NRS-T3: Pain intensity at 24 h postoperatively, X: Mean, SD: Standard deviation.

**Table 5:** Association of Surgical Fear and Pain with Recovery: Multiple Regression Analysis.

		Unstandardized Coefficients		Standardized Coefficients	t	Sig.	95.0% Confidence Interval for B		Collinearity Statistics	
		B	Std. Error	Beta			Lower Bound	Upper Bound	Tolerance	VIF
Model for Physical comfort	(Constant)	47.598	3.218		14.791	0.000	41.196	54.000		
	*SFQ*	−0.154	0.044	−0.364	−3.466	**0.001**	−0.242	−0.066	0.953	1.049
	NRS-T0	0.186	0.229	0.086	0.810	0.420	−0.270	0.641	0.927	1.079
	NRS-T1	0.309	0.238	0.138	1.296	0.199	−0.165	0.783	0.935	1.070
	NRS-T2	−0.212	0.323	−0.071	−0.657	0.513	−0.855	0.431	0.890	1.123
	NRS-T3	0.296	0.365	0.091	0.810	0.420	−0.430	1.022	0.839	1.192
	F = 2.588; *p* = 0.032; R^2^ = 0.369; Adjusted R^2^ = 0.084									
Model for Emotional state	(Constant)	42.877	2.567		16.701	0.000	37.770	47.984		
	*SFQ*	−0.180	0.035	−0.488	−5.083	**0.000**	−0.251	−0.110	0.953	1.049
	NRS-T0	−0.165	0.183	−0.088	−0.901	0.370	−0.528	0.199	0.927	1.079
	NRS-T1	−0.167	0.190	−0.085	−0.879	0.382	−0.545	0.211	0.935	1.070
	NRS-T2	0.094	0.258	0.036	0.365	0.716	−0.419	0.607	0.890	1.123
	NRS-T3	−0.270	0.291	−0.095	−0.926	0.357	−0.849	0.309	0.839	1.192
	F = 6.322; *p* < 0.001; R^2^ = 0.527; Adjusted R^2^ = 0.234									
Model for Physical independence	(Constant)	18.822	1.845		10.199	0.000	15.151	22.493		
	*SFQ*	−0.050	0.025	−0.195	−1.951	0.054	−0.100	0.001	0.953	1.049
	NRS-T0	0.237	0.131	0.183	1.807	0.074	−0.024	0.499	0.927	1.079
	NRS-T1	0.205	0.137	0.151	1.498	0.138	−0.067	0.477	0.935	1.070
	NRS-T2	−0.391	0.185	−0.218	−2.110	**0.038**	−0.760	−0.022	0.890	1.123
	NRS-T3	−0.414	0.209	−0.211	−1.980	0.051	−0.831	0.002	0.839	1.192
	F = 4.654; *p* < 0.001; R^2^ = 0.470; Adjusted R^2^ = 0.174									
Model for Patient support	(Constant)	33.600	1.693		19.846	0.000	30.232	36.968		
	*SFQ*	−0.096	0.023	−0.422	−4.105	**0.000**	−0.142	−0.049	0.953	1.049
	NRS-T0	0.080	0.121	0.069	0.661	0.510	−0.160	0.319	0.927	1.079
	NRS-T1	0.109	0.125	0.091	0.873	0.385	−0.140	0.359	0.935	1.070
	NRS-T2	−0.094	0.170	−0.059	−0.555	0.580	−0.433	0.244	0.890	1.123
	NRS-T3	0.105	0.192	0.060	0.547	0.586	−0.277	0.487	0.839	1.192
	F = 3.421; *p* = 0.007; R^2^ = 0.415; Adjusted R^2^ = 0.122									
Model for Pain	(Constant)	32.598	1.716		18.998	0.000	29.185	36.011		
	*SFQ*	−0.114	0.024	−0.475	−4.817	**0.000**	−0.161	−0.067	0.953	1.049
	NRS-T0	−0.111	0.122	−0.091	−0.912	0.365	−0.354	0.132	0.927	1.079
	NRS-T1	0.097	0.127	0.076	0.763	0.447	−0.156	0.350	0.935	1.070
	NRS-T2	−0.176	0.172	−0.104	−1.021	0.310	−0.519	0.167	0.890	1.123
	NRS-T3	−0.030	0.195	−0.016	−0.155	0.877	−0.417	0.357	0.839	1.192
	F = 5.159; *p* < 0.001; R^2^ = 0.489; Adjusted R^2^ = 0.193									
Model for QoR-40 total score	(Constant)	183.393	7.218		25.407	0.000	169.036	197.749		
	*SFQ*	−0.617	0.100	−0.563	−6.170	**0.000**	−0.816	−0.418	0.951	1.051
	NRS-T0	−0.005	0.517	−0.001	−0.010	0.992	−1.035	1.024	0.924	1.082
	NRS-T1	0.360	0.538	0.062	0.670	0.505	−0.709	1.430	0.934	1.071
	NRS-T2	−1.240	0.730	−0.160	−1.699	0.093	−2.692	0.212	0.890	1.123
	NRS-T3	−0.633	0.817	−0.075	−0.776	0.440	−2.257	0.991	0.837	1.195
	F = 8.663; *p* < 0.001; R^2^ = 0.586; Adjusted R^2^ = 0.303									

B: Unstandardized regression coefficient; Beta (β): Standardized regression coefficient; CI: Confidence Interval; t: Independent samples *t*-test; F: ANOVA test statistic; NRS: Numerical Rating Scale; QoR-40: Quality of Recovery-40; SFQ: Surgical Fear Questionnaire; Std. Error: Standard Error; No multicollinearity was detected (VIF < 5, tolerance > 0.20).

## Data Availability

The original contributions presented in this study are included in the article. Further inquiries can be directed to the corresponding authors.

## Abbreviations

The following abbreviations are used in this manuscript:

QoR-40	Quality of Recovery Scale
SFQ	Surgical Fear Questionnaire
NRS	Numeric Rating Scale
STROBE	Strengthening the Reporting of Observational Studies in Epidemiology
ASA	American Society of Anesthesiologists
TKA	Total Knee Arthroplasty
THA	Total Hip Arthroplasty
NRS-T0	Preoperative pain intensity
NRS-T1	Pain intensity prior to the postoperative first analgesic administration
NRS-T2	Pain Intensity at 12 h postoperatively
NRS-T3	Pain intensity at 24 h postoperatively
VIF	Variance inflation factor
